# HaplotypeCN: Copy Number Haplotype Inference with Hidden Markov Model and Localized Haplotype Clustering

**DOI:** 10.1371/journal.pone.0096841

**Published:** 2014-05-21

**Authors:** Yen-Jen Lin, Yu-Tin Chen, Shu-Ni Hsu, Chien-Hua Peng, Chuan-Yi Tang, Tzu-Chen Yen, Wen-Ping Hsieh

**Affiliations:** 1 Department of Computer Science, National Tsing Hua University, Hsinchu, Taiwan; 2 Institute of Statistics, National Tsing Hua University, Hsinchu, Taiwan; 3 Department of Computer Science and Information Engineering, Providence University, Taichung, Taiwan; 4 Department of Resource Center for Clinical Research, Chang Gung Memorial Hospital, Taoyuan, Taiwan; 5 Head and Neck Oncology Group, Chang Gung Memorial Hospital, Taoyuan, Taiwan; 6 Nuclear Medicine and Molecular Imaging Center, Chang Gung Memorial Hospital, Taoyuan, Taiwan; New Jersey Institute of Technology, United States of America

## Abstract

Copy number variation (CNV) has been reported to be associated with disease and various cancers. Hence, identifying the accurate position and the type of CNV is currently a critical issue. There are many tools targeting on detecting CNV regions, constructing haplotype phases on CNV regions, or estimating the numerical copy numbers. However, none of them can do all of the three tasks at the same time. This paper presents a method based on Hidden Markov Model to detect parent specific copy number change on both chromosomes with signals from SNP arrays. A haplotype tree is constructed with dynamic branch merging to model the transition of the copy number status of the two alleles assessed at each SNP locus. The emission models are constructed for the genotypes formed with the two haplotypes. The proposed method can provide the segmentation points of the CNV regions as well as the haplotype phasing for the allelic status on each chromosome. The estimated copy numbers are provided as fractional numbers, which can accommodate the somatic mutation in cancer specimens that usually consist of heterogeneous cell populations. The algorithm is evaluated on simulated data and the previously published regions of CNV of the 270 HapMap individuals. The results were compared with five popular methods: PennCNV, genoCN, COKGEN, QuantiSNP and cnvHap. The application on oral cancer samples demonstrates how the proposed method can facilitate clinical association studies. The proposed algorithm exhibits comparable sensitivity of the CNV regions to the best algorithm in our genome-wide study and demonstrates the highest detection rate in SNP dense regions. In addition, we provide better haplotype phasing accuracy than similar approaches. The clinical association carried out with our fractional estimate of copy numbers in the cancer samples provides better detection power than that with integer copy number states.

## Background

Copy number alterations are segments of nucleotide sequences that are amplified or deleted compared to the regular ploidy number of certain organism. Such structural abnormalities of chromosomes may be caused by germline alterations, the copy number variations (CNV), or acquired in somatic mutations, which is specifically termed copy number aberration (CNA). Different patterns of the copy number alteration may give rise to varying phenotypes among individuals or may be the key of understanding disease susceptibility. Copy number variation have been reported to be associated with Alzheimer’s disease (AD) [1], Crohn’s disease [2], autism [3], Parkinson’s disease [4], and Schizophrenia [5]. In contrast, copy number aberrations were mostly associated with various cancer types [6], [7] [8]. However, copy number alterations spread over the human genome even in normal individuals [9]. Most CNVs/CNAs are neutral events that do not affect any phenotypes. Understanding the distribution of the copy number events among normal individuals can help to discover the abnormalities that actually result from copy number changes. Hence, identifying the accurate position and the type of copy number changes in the human genome is currently a critical issue. Many technologies have been developed to detect copy number alteration, including fluorescence in situ hybridization (FISH), comparative genomic hybridization (CGH), array comparative genomic hybridization (aCGH), SNP array, and massive parallel sequencing. In this study, we focus on the information extracted from the Affymetrix SNP 6.0. Other platforms that retrieve allele-specific signals can fit into the same framework for copy number detection.

Various algorithms are designed to detect regions of copy number variation with SNP array data [10]–[18]. The basic ideas behind these algorithms can be roughly categorized into segmentation-based, HMM-based, model-based strategies and others that combine with any of the aforementioned three. The first group of detection algorithms are segmentation-based methods, originally designed for array Comparative Genomic Hybridization (aCGH). Popular packages include Circular Binary Segmentation [19], and GADA [14]. The first step of this category involves deciding the segmentation point of copy number variation regions according to a criterion or statistical tests. The estimation of copy numbers is provided as a second step. This type of analysis can only provide the total copy numbers while ignore the allele specific information.

The algorithms based on HMM include QuantiSNP [11], PennCNV [10], GenoCN [12], PICNIC [20] and cnvHap [13]. The transition model of the first three algorithms account for the distance between neighboring loci; the longer the distance between any two loci, the more likely a state change will occur between them. QuantiSNP, PennCNV, PICNIC, and GenoCN can provide allele-specific copy numbers (ASCN), and cnvHap can further provide haplotypes composed of allelic copy number change on the same chromosomes. However, the copy numbers are pre-specified as part of the hidden states and hence can only consider integer numbers.

Another group of algorithms are model-based methods including Copy Number Analysis with Regression And Tree [15] (CARAT), Probe-Level Allele-Specific Quantitation [16] (PLASQ). They can infer fractional allele-specific copy numbers (ASCN) on tumor samples. CARAT and PLASQ methods require normal samples to train the rules. In the end, the copy numbers are smoothed across SNPs within each copy number alteration region. PLASQ can also infer parent-specific copy number (PSCN) based on the assumption that copy number and probe intensity are approximately linearly correlated on a log-log scale.

Several recent researches studied allele-specific copy numbers. The studies of Olshen et al [21]., Chen et al [22] and Tumor Aberration Prediction Suite (TAPS) [23] are among this category. TAPS adopts Circular Binary Segmentation (CBS) to detect the CNV region. It provides allelic imbalance ratio calculation, and copy number calling. PSCN [22] and PSCBS [21] can further infer the parent specific copy numbers. That is, they provide the total copy number on each one of the two chromosomes for the CNV regions they detect. They reported simultaneous change-point analysis of paired allele-specific CNV data [21] and log of total copy number [22]. Both platforms generate contiguous copy number measures along ordered chromosomal location. However, the algorithm was developed to solve the issue of copy neutral LOH and did not function with reasonable accuracies for a general purpose CNV detection so it is not included in our comparison. Detailed observations are provided Table S3 in File S4 and Figure S1–S6 in File S4 to support our suggestion.

Some methods require multiple steps to derive the copy number variation. Birdsuite [18] is a four-stage algorithm to integrate copy numbers and genotype calling on CNV regions. The third step of Birdsuite is also based on HMM to infer copy numbers on CNV regions with the results of the first two steps. Its final step combines all information obtained from the preceding steps to estimate the allele-specific copy number calls for the CNV regions. COKGEN [17], by contrast, uses an optimization based approach that depends on an objective function and a searching algorithm. Simulated annealing is used to iteratively obtain the most accurate CNV calls. However, both of the strategies provide discrete copy numbers. Therefore, those strategies cannot provide accurate information of the primary tumor cells, which are heterogeneous with mixtures of normal and cancerous cells. To solve this issue, the Allele-specific copy number analysis of tumors (ASCAT) [24] provides whole-genome allele-specific copy number and construct a genome-wide map of loci with alleles that were preferentially lost, pointing to candidate genes that may drive cancer development.

As mentioned above, cnvHap provide chromosome specific haplotypes. This type of studies include MOCsphaser [25] and the study of Huang et al. [26]. However, MOCsphaser requires the CNV regions and the total number copy numbers to be known in advance. The study of Huang et al. re-organizes the known haplotypes from reference samples into a pre-specified CNV region of known total copy numbers. They both need to know CNV regions in advance.

Most of the aforementioned methods provide certain advantages to others while they still lose information in some manner. We describe a method, termed HaplotypeCN that adopts the Hidden Markov Model as the basic framework. This method can provide fractional allele-specific copy numbers, detect CNV regions and construct phased haplotypes with haplotype-specific copy numbers at the same time. To be more specific, HaplotypeCN can reconstruct the haplotypes composed of allelic copy number change on the same chromosomes. Haplotypes consist of alleles of adjacent loci and are transmitted together from a single parent. They are natural genetic units produced by the recombination mechanism. It has been reported to help improve the power of detection in the genome-wide association under some circumstances. Haplotypes can be inferred from genotypes. This step is called phasing. Correctly detecting CNA haplotypes helps to identify the chromosomes that are more susceptible to structure variation. The formation of tumor is a process of accumulation of mutations and it is an evolutionary process [27]. Recurrent amplicons were observed in different types of somatic mutation [28], [29]. Certain haplotypes might be disproportionately shared by tumors [30], [31]. There is no way to comprehensively study the occurrences if we cannot accurately construct the haplotypes. The challenge of haplotype phasing for diploid genomes has drawn considerable attention and achieved significant progress. We did not adopt the common sliding window methods to determine the haplotype length. The haplotype lengths are dynamically determined based on the variable-length Markov chains (VLMC) model [32].

The proposed HaplotypeCN is compared with several popular algorithms including PennCNV, QuantiSNP, genoCNV, COKGEN, and cnvHap because they have software freely available and can be applied on Affymetrix SNP 6.0 arrays. The performance is evaluated on the 270 HapMap samples that have been genotyped on Affymetrix 6.0 platforms. Copy number variation regions for those samples have been reported in two studies using different technology and are treated as, benchmark events for concordance evaluation. In this study, we also provide simulation data to compare the haplotype phasing results between HaplotypeCN and cnvHap. The proposed HaplotypeCN can achieve better accuracy than cnvHap.

## Materials and Methods

### Ethics Statement

Written informed consent was obtained from all participants. This study was approved by the Chang Gung Memorial Hospital Human Research Ethics Committee (Protocol number 98-3645B).

### Initial Input and Allelic States

The algorithm was conducted iteratively with two major steps: (I) the probabilistic haplotype tree construction and merging, and (II) the haplotype state recognition from the array intensity data based on two independent haplotype trees and the emission probability model. Before the iteration of the two steps, the initiation requires a rough guess of the genotypes for each sample and it can be easily derived from any genotype calling algorithm. We use the probe intensity summary and the genotype calls from Birdseed, the second step of the Birdsuite package [18], as input of our program. No information about amplification or deletion is provided at this stage. When there is no call from Birdseed, we impute the genotype with the smallest Mahalanobis distance to the three models of AA, AB and BB. Every locus can be any one of the three genotypes, AA, AB or BB. The data will be used to initiate the haplotype tree construction.

Six states are defined for each SNP locus on a single chromosome. They are A^−^, A, A^+^, B^−^, B, and B^+^ respectively representing deletion of A allele, one copy of A allele, amplification of A allele, deletion of B allele, one copy of B allele and amplification of B allele. The normal state A and B can transit to all six states. The states of loss (A^−^ or B^−^) can only transit to either states of loss, or transit to the normal states (A or B) at the neighboring locus. By contrast, the states of gain (A^+^ or B^+^) can only transit to either states of gain, or transit to a normal states (A or B). Consequently, only 28 possible haplotypes exist, rather than 6×6 = 36 haplotypes between any two consecutive loci. The restriction is based on a reasonable assumption that acquiring both DNA loss and gain at the adjacent regions is less likely; although it is possible biologically, we do not expect it to be common events. With current computational power in contrast to the enormous dimension of data, spending the degrees of freedom on rare events and sacrifice the estimation accuracy is of less interest.

### Estimation of Four Haplotype Proportion for All Pairs of Consecutive Loci

Starting from the genotypes derived from Birdseed, we construct the initial transition probability between all transferrable allelic states at every pair of loci by multiplying a state-dependent scaling factor with the haplotype proportions estimated from the genotype counts. The nine genotype counts between loci *j* and *j+1* in a sample are 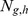, where 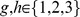 and the genotypes AA, AB and BB are coded as 1, 2 and 3 respectively in either locus.

We first estimate the proportions for the four major haplotypes A−A, A−B, B−A and B−B from the genotypic data at each pair of consecutive loci. Eight of the nine genotypic combinations can be resolved for the haplotypes. For example, the genotype (AA)_j_(AB)_j+1_ between loci j and j+1 is counted for N_1,2_ times and each individual with this genotype carries a haplotype A−A and a haplotype A−B. The only genotype with more than two sets of possible haplotype combinations is (AB)_j_(AB)_j+1_, which is heterozygous at both loci. The haplotype combination for (AB)_j_(AB)_j+1_ can be either A−A/B−B or A−B/B−A. We assume the proportion of the haplotype pair A−A/B−B in the N_2,2_ samples to be 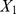 and estimate it in an iterative process with the estimation of all the haplotype probabilities. The proportion of the four haplotypes can be estimated as equation (1) with the initial value of 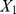 set to 0.5. The value for 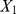 is then updated with equation (2). The iteration between equations (1) and (2) will stopped when the difference of 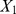 between any two consecutive iterations is smaller than 10^−5^. The derived estimates of 
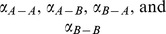
 are used as our input to the estimation of transition probability.
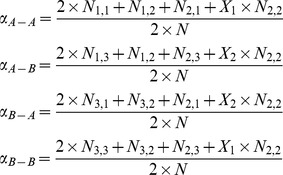
(1)

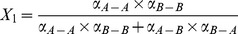
(2)


The iterative estimation for the haplotype proportions is only required at the initiation step. Because we do not have information about allelic amplification or deletion from the Birdseed genotypic data, haplotypes consisting of allelic states with amplification or deletion are implicitly included in the above four haplotypes. That is, the proportion of haplotype A−A should also consist of proportions of A−A^+^, A−A^−^, A^+^−A, A^+^−A^+^, A^−^−A and A^−^−A^−^. Those seven haplotypes belong to the same set and are denoted as haplotype set A−A. Similarly, haplotype sets A−B, B−A and B−B all consist of seven haplotypes. After proceeding through the following steps with one global iteration, the haplotype phase for the two chromosomes will be resolved throughout the whole genome and the proportion of the 28 haplotypes between any two loci can be derived. We add the proportions of seven haplotypes of the same haplotype set to derive the four group proportions, 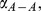 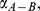 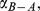 and 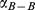
_._

Because the haplotype probability represents the joint probability between two loci, the conditional probability is calculated as
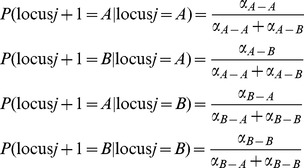
(3)


### Haplotype Tree Construction with Dynamic Branch Merging

**Figure 1:**
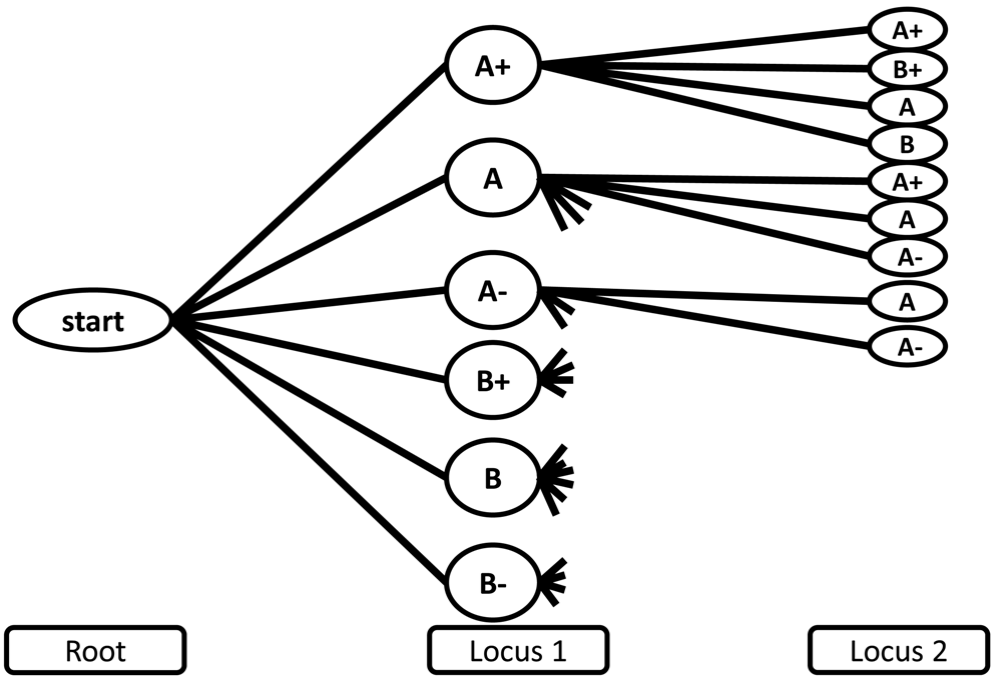
A haplotype tree with only two loci.

The first step of our global iteration involves constructing a tree to represent the state transition of either chromosome. The haplotype tree we construct is a probabilistic tree in the sense that the branches of the tree represent the transition probabilities between states at consecutive loci. Starting from a root node with no physical meaning, the haplotype tree branches to six nodes representing the six allelic states defined previously for the first locus. Each of the six nodes branch further to six or four nodes, depending on the current states according to the restriction we place between amplification and deletion states (Figure 1). With just a few branching steps, it becomes a gigantic tree that requires incredibly large computations. Fortunately, haplotype construction from genotypic data has been studied extensively with many algorithms, such as Variable Length Markov Chain (VLMC), PHASE and fastPHASE, which are able to handle this problem efficiently [33], [34]. We adopt the Variable Length Markov Chain (VLMC) proposed by Browning [32] to merge the branches dynamically. The merged nodes could be positions with higher recombination rates, and the stretch between any two consecutive merged points loosely represents a haplotype block.

**Figure 2:**
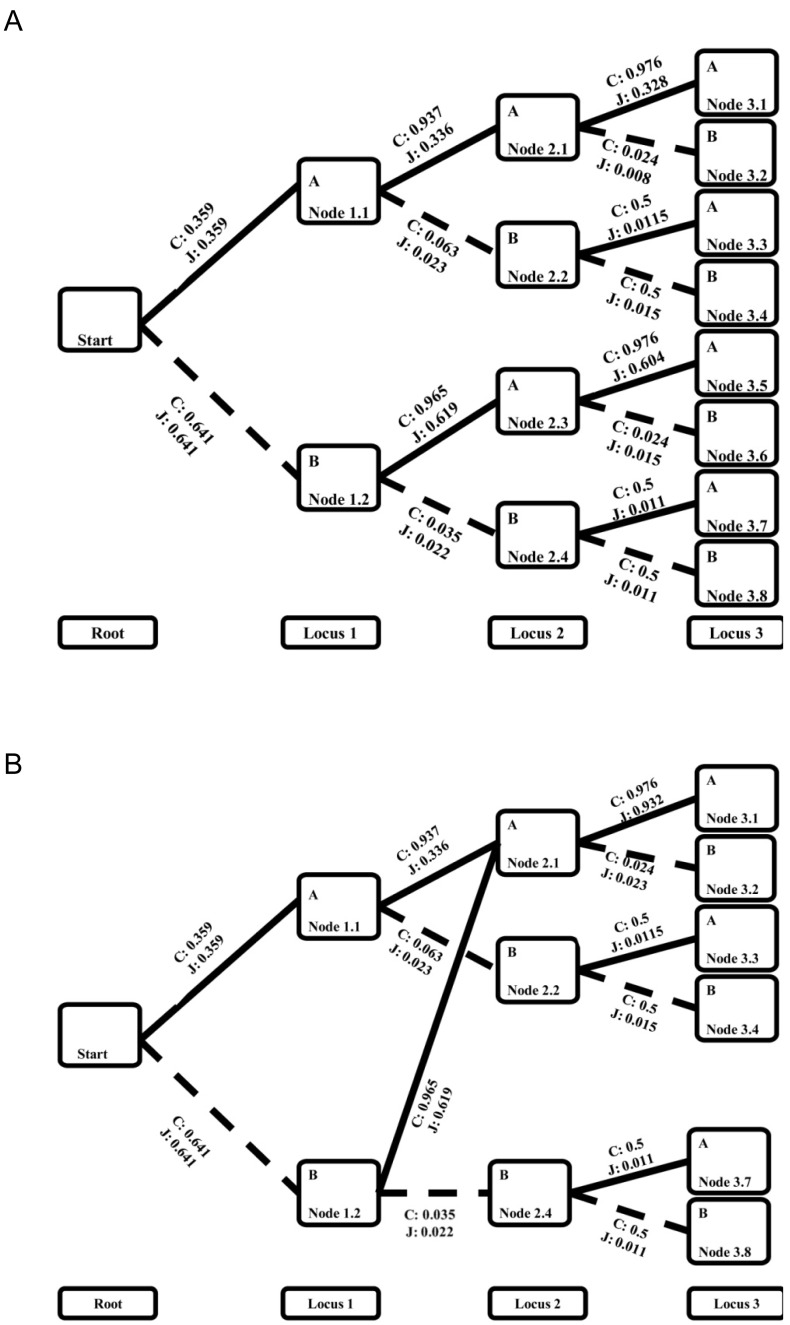
Tree merging example. Each branch is attached with a conditional probability *C* from the left node to the right node and a joint probability *J* of the haplotype between the left node and the right node. Panel (a) presents a tree before merging Node 2.1 and Node 2.3, and panel (b) presents the tree after merging.

The merging step was performed on the haplotype sets instead of on all the branches in Figure 1. Figure 2 depicts an actual example. The joint probability α’s calculated in Equation (1) are indicated as J, and the conditional probabilities calculated in Equation (3) are indicated as C in Figure 2. The binary tree in Figure 2 was constructed for only two alleles at each locus without considering any amplification or deletion states. The aberrant states are implicitly included and will be branched out later. The nodes with similar branching probability to the next locus are merged. Using the conditional probabilities calculated in Equations (3), we derived a similarity score for each pair of nodes at the same locus by calculating the maximal absolute difference of conditional probabilities between the parallel descendants. For example, to compare Node 2.1 and 2.3 in Figure 2(a), the difference is taken between the conditional probability from Node 2.1 to allele A (Node 3.1) and the conditional probability from Node 2.3 to allele A (Node 3.5). It is |0.976−0.976| = 0. The difference is also taken between Node 2.1 and 2.3 to Node 3.2 and Node 3.6 respectively. It is |0.024−0.015| = 0.009. The similarity score between Node 2.1 and 2.3 is the maximum of the two and is 0.009. Let *M*_2.1_ and *M*_2.3_ represent the numbers of haplotype counts for the haplotypes starting from the root node and ending in Node 2.1 and Node 2.3. The threshold for the similarity score is set at 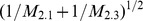, which is roughly twice the standard deviation of the absolute difference when the transition is purely guided by a random process [32]. The pairs of nodes with similarity scores smaller than their corresponding threshold are candidates to be merged, and the pair with the smallest similarity score is merged first. Figure 2(b) demonstrates the tree with Node 2.1 and Node 2.3 merged from the tree in Figure 2(a). Their descendants are merged in parallel with probability adjustment described in the next paragraph. Similarity scores will then be updated again between the new node and all the other nodes at the same locus. The most similar pair will be merged if the similarity score is below the threshold. The iteration stops when no more pairs can be merged or when only one node remains. The merging step is performed sequentially from the first locus to the final one. According to the simulation in Browning’s study [32], 90% of the similarity scores are lower than the threshold and the nodes are merged.

The conditional probabilities from the merged node to the descendants are essentially the weighted average of the conditional probabilities of each individual node before the merge. The weights are the haplotype counts for each original node. For example, the conditional probability P(A_Node3.1_ | A_Node2.1_) in the merged node of Figure 2b is calculated as [*M*_2.1_P(A_Node3.1_ | A_Node2.1_) + *M*_2.3_P(A_Node3.5_ | A_Node2.3_)]/(*M*_2.1_+ *M*_2.3_) in the nodes of Figure 2a, and the conditional probability P(B_Node3.2_ | A_Node2.1_) in the merged node in Figure 2b is calculated as [*M*_2.1_P(B_Node3.2_ | A_Node2.1_) + *M*_2.3_P(B_Node3.6_ | A_Node2.3_)]/(*M*_2.1_+ *M*_2.3_) in the nodes of Figure 2a. The results are displayed in Figure 2b.

**Figure 3:**
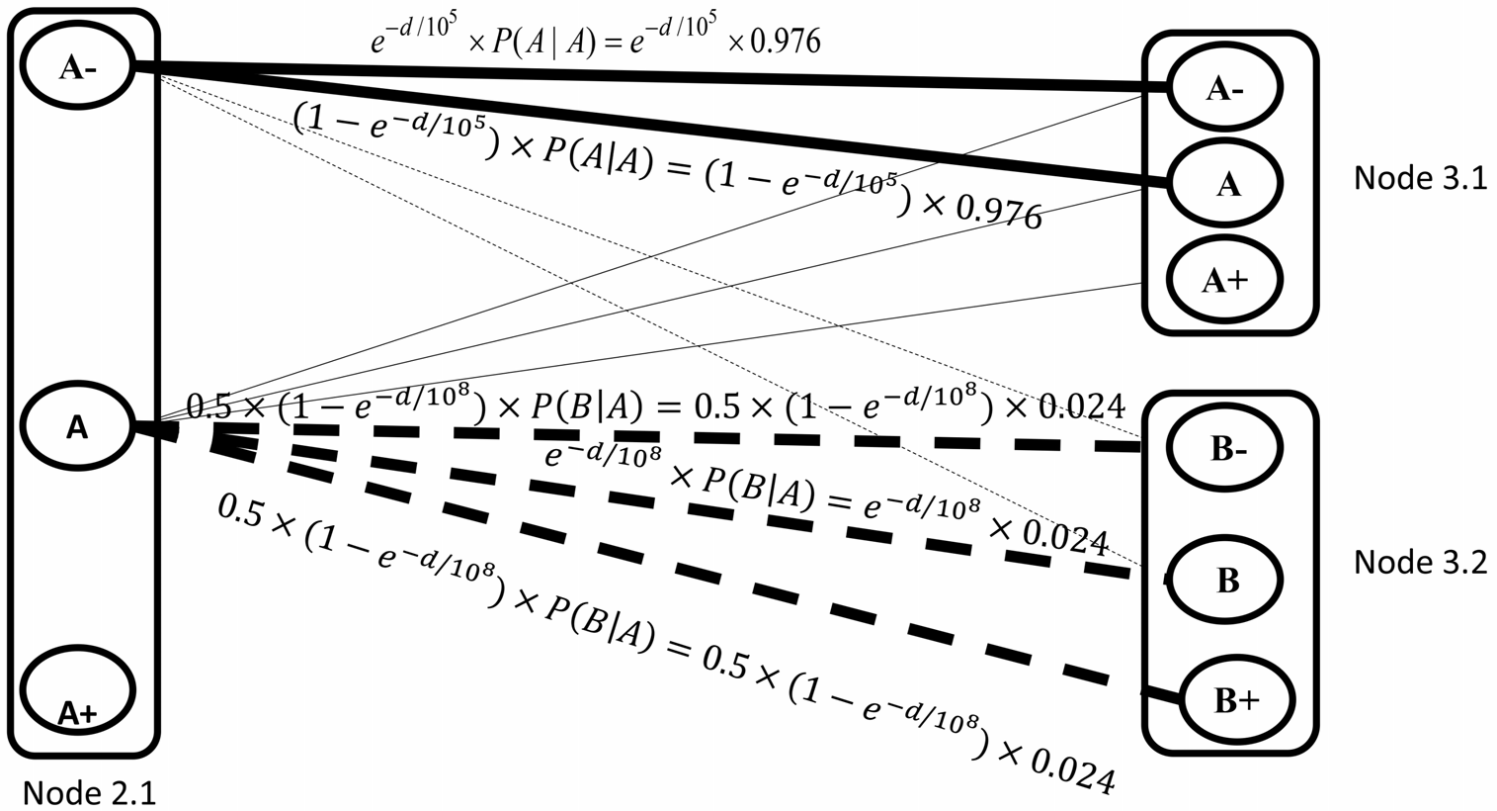
Branching to derive the amplification or deletion state. The thick lines are marked with the formula of transition probabilities, while the thin lines can be calculated accordingly.

After the tree merging process at the haplotype set level, the tree is branched out for all the amplification or deletion states. That is, the conditional probabilities are distributed to the transition probabilities for haplotypes in the same haplotype set by multiplying an empirically derived factor. In this step, we consider the adjustment with distance effect according to the assumption that the longer the distance between the two loci is, the less likely the copy number status will stay at the same condition. Similar to previous studies [10], [11], we assume that the decreasing rates follow an exponential function of distance, 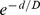, where *d* is the distance in terms of nucleotides between the neighboring loci (10) and 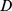 decides the exponentially decreasing rate. Hence, the increasing rate is the complement 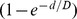. The transition probabilities are defined as follows.
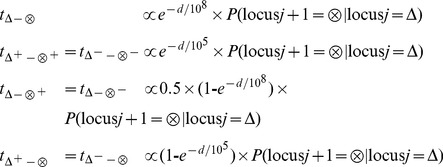
(4)where Δ or 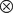 can be either A or B and the superscript + or – stands for the amplification or deletion state. For example, the conditional probability P(A | A) is distributed to the transition probabilities 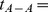
updated P(A | A), 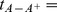
P(A^+^ | A) and 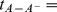
P(A^−^ | A) with proportion 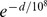, 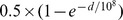 and 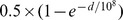. The transition probabilities 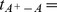
P(A | A^+^) and 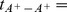
P(A^+^ | A^+^) share the same conditional total P(A | A) with proportion 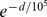
and 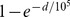. Similarly, the transition probabilities 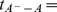
P(A | A^−^) and 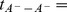
P(A^−^ | A^−^) also share the same conditional total P(A | A) with proportion 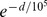 and 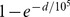. Figure 3 provides a branching example for the merged Node 2.1 in Figure 2b. The number D is selected as 10^8^ and 10^5^ for the normal allelic state and the variant copy number state, respectively. The assumption behind the setting lies on the observation that the stretch of a normal genomic sequence is considerably longer than the aberrant sequences with either amplification or deletion. The number D is set as fixed in our implementation based on the empirical studies of several datasets.

### Emission Probability

Observations derived from SNP arrays are the probe intensities summarized for the signal of Allele A and Allele B at each locus. Hence, each observation is a two dimensional data with paired signal (s^A^, s^B^) at each locus. Our recognition process is based on the Hidden Markov Model (HMM). The transition states are the 36 genotypes (A^−^, A, A^+^, B^−^, B, B^+^)×(A^−^, A, A^+^, B^−^, B, B^+^) at each locus and are constructed from an independent multiplication of two haplotype trees.

**Figure 4:**
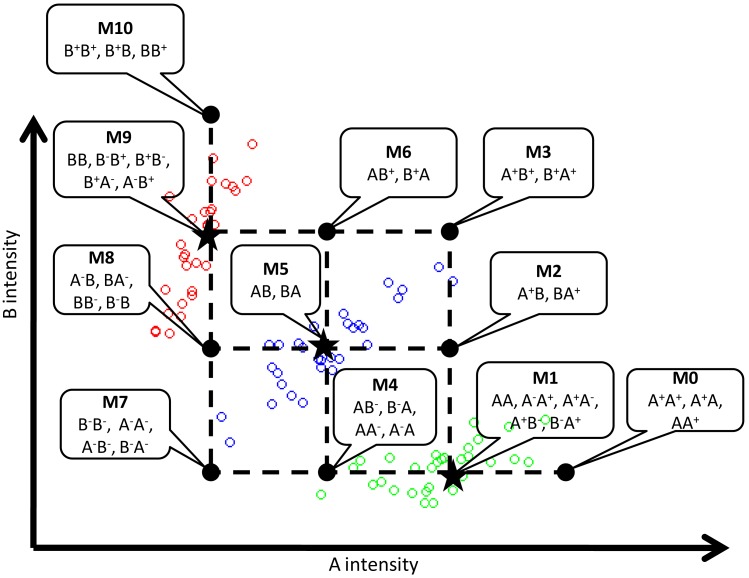
Centers of the eleven emission probability models. The green, blue and red circles represent the observed samples with genotype AA, AB and BB according to the genotypes identified by Birdseed. The stars are the centers of the three initial groups. All the other centers are assigned according to the grids passing through the stars. M0 and M10 are extended from M1 and M9 so that distance between centers of M1 and M0 is equal to the distance between centers of M1 and M4, and the distance between centers of M9 and M10 is equal to the distance between centers of M9 and M8.

The emission probability model is assumed to be a bivariate normal distribution for each genotypic state. At the initiation step, the models at each locus are seeded with three emission probability models for genotype AA, AB and BB from the genotype-calling module of Birdseed, in which the package provides the mean vector and the covariance matrix for the bivariate normal distribution. The three models are extended to 11 models according to the grouping in Table 1 and Figure 4. The model parameters are assigned in Table 2. Each group collects genotypes with close signal distribution and they share the same emission probability model. The parameters of each model are updated after each iteration of recognition. The copy number estimation and the procedure of parameter update are described in the next section.

**Table 1:** **Groups of genotypic states with respective characteristics.**

Emission probability model	Description	Genotypic States
M0	Allele A is present in both chromosome with total copy number greater than two, and Allele B is not present in either chromosome.	A^+^A^+^,A^+^A, AA^+^
M1	Allele A is present in at least one chromosome with total copy number close to two, and Allele B is barely present in either chromosome.	AA,A^−^A^+^, A^+^A^−^, A^+^B^−^, B^−^A^+^
M2	Allele A is present in one chromosome with copy number greater than one, and Allele B is present in the other chromosome with one normal copy.	A^+^B, BA^+^
M3	Both chromosomes present events of gain.	A^+^B^+^, B^+^A^+^
M4	Allele A is present in one chromosome with one normal copy, and Allele A or B is present in the other chromosome with less than one copy.	AB^−^,B^−^A,AA^−^,A^−^A
M5	Allele A is present in one chromosome with one normal copy, and Allele B is present in the other chromosome with one normal copy. It is a normal heterozygote.	AB, BA
M6	Allele B is present in one chromosome with copy number greater than one, and Allele A is present in the other chromosome with one normal copy. This is a symmetric model of M2 with flipping alleles.	AB^+^, B^+^A
M7	Both chromosomes present events of loss.	B^−^B^−^,A^−^A^−^,A^−^B^−^, B^−^A^−^,
M8	Allele B is present in one chromosome with one normal copy, and Allele A or Bis present in the other chromosome with less than one copy. This is a symmetric model of M4.	A^−^B, BA^−^, BB^−^, B^−^B
M9	Allele B is present in at least one chromosome with total copy number close to two and, allele A is barely present in either chromosome. This is a symmetric model of M1.	BB, B^−^B^+^, B^+^B^−^, B^+^A^−^, A^−^B^+^
M10	Allele B is present in both chromosome with total copy number greater than two, and allele A is not present in either chromosome. This is a symmetric model of M0.	B^+^B^+^, B^+^B, BB^+^

**Table 2:** **The mean vector and variance component assignment of the emission probability models at the initiation step.**

Emission probability model (M)	Mean (Mean of A signal, Mean of B signal)	Covariance (Variance of A signal, Covariance between A and B signal, Variance of B signal)
**M0**	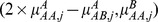	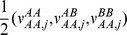
**M1**	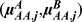	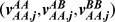
**M2**	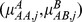	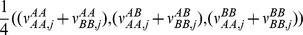
**M3**	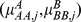	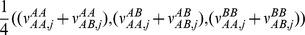
**M4**	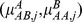	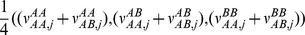
**M5**	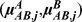	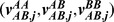
**M6**	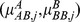	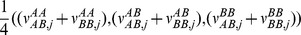
**M7**	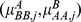	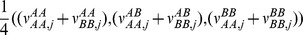
**M8**	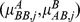	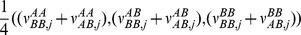
**M9**	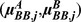	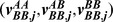
**M10**	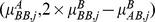	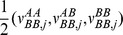

The assignments of M1, M5 and M9 are extended to all the other models.

### Detection of Copy Number Alteration, Haplotype Phasing, Model Parameter Update, and Copy Number Estimation

With the above emission probability models and the transition probability, the best sequence of hidden states that represent the combination of the two phased haplotypes is derived by the Viterbi algorithm [35]. Because the number of all genotypic states is too large to handle, we consider only the group of genotypes with the smallest Mahalanobis distance from the model center, 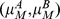, to the observed intensity pair 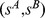 at each SNP locus. The distance measure is calculated as 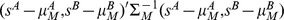 where Σ*_M_* is the covariance matrix of model *M*, and the model with the smallest distance to 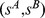 is selected. The haplotypes are phased only for the genotypes in the selected group. When all the 36 combinations are considered, the computation load is very heavy. In order to reduce the computational time, we use Mahalanobis distance to group genotypes. For each group we use Viterbi algorithm to choose the best genotype. The shaded region was designed to reduce the false positives. Compared with the full model using 36 combinations, this heuristic rule does not reduce the detection accuracy a lot. We will explain it with more details later.

**Figure 5:**
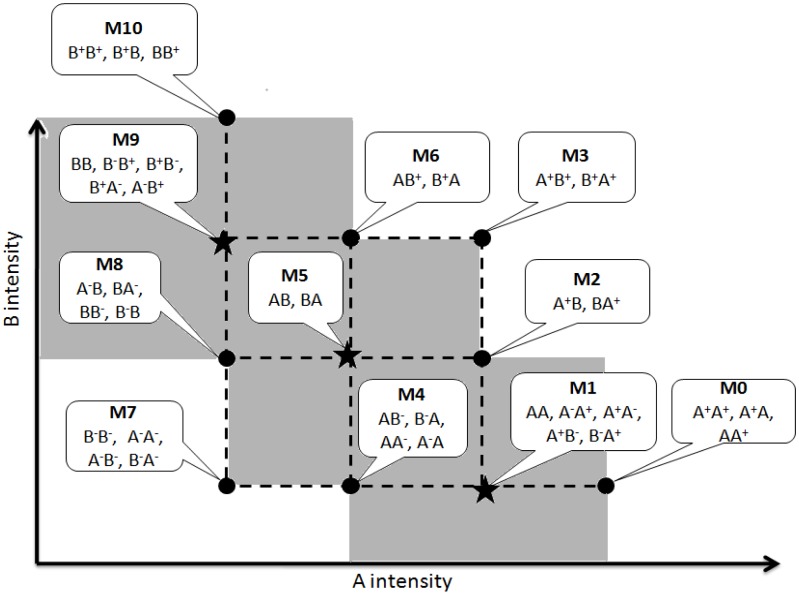
The shaded region applied to filter unreliable CNV prediction.

After the haplotype-phasing step, we have potential copy number alteration regions generated with genotype calls for allelic gain or loss at each SNP locus. Further filtering criterions are applied on the regions to avoid overly fragmented results. Firstly, any copy number alteration region with a single SNP will be excluded. Secondly, If only one or two SNPs exist between two predicted regions of the same direction of copy number change (both plus or both minus states), the two copy number alteration regions will be merged into one by including the middle loci that were not reported in the beginning. Thirdly, we mark SNPs within copy number alteration calls as low confidence if their signals 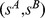 fall into the shaded region of Figure 5. Because the region includes most of the probe signals from the three normal genotypes, AA, AB and BB, a predicted copy number alteration region consisting of a moderate number of SNPs in this shaded region indicates only mild deviation from the normal sequence and, hence, is removed to reduce false positives. If a predicted region contains more than 4/5 of the SNPs marked as low confidence, the region is excluded. When a predicted copy number alteration region consists of less than four SNPs, none of the intensity pairs are allowed to be in the shaded region.

Although all of our emission models are extended from M1, M5 and M9 at the initiation, parameters of all the eleven models need to be updated in the following iterations. The mean vector is updated by averaging the SNP signals belonged to each model across samples and the sample covariance matrix is calculated accordingly. When the samples belonged to a specific group are less than two, we will use the extension from new M1, M5, and M9 to update the mean and covariance as the initiation step. The transition probabilities are updated after the filtering step. The conditional probabilities in Equation (3) are now calculated according to the phased haplotype counts at all pairs of loci.

Because the haplotypes throughout the genome are phased in the recognition step, the genotypic states are assigned to each SNP locus. The states defined in the HMM process do not specify the explicit copy numbers for each individual at each SNP locus. They are estimated at a post-processing step by interpolation and extrapolation relative to the centers of model M1, M5, and M9. Fractional numbers are provided because our algorithm aims at determining copy number aberration in tumor samples, where the heterogeneity of cells usually leads to different copy number status among cells. A fractional copy number estimation can provide a quantitative measurement of how large the aberration is in the sample under evaluation.

The allele-specific copy number 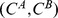 for each SNP locus is estimated as
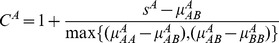
(5)

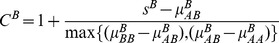



The estimation of copy number is based on interpolation or extrapolation from the heterozygous group, which has one copy for either the *A* allele or the *B* allele. Ideally, the difference between 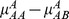 and 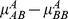 should be similar and so is the difference of 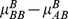 and 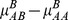. When there is significant difference, the larger one is used as the divisor in equation (5) as a conservative approach. The total copy number for each predicted copy number alteration region is calculated by averaging the copy number estimates of all the containing SNPs and is provided in our package.

To summarize the outputs of HaplotypeCN, we use the result from the real data analysis as an example. In the first step, HMM performs as a classifier to retrieve the regions with CNV events and separate the amplification from the deletion status. For example, we can produce haplotypes at this stage as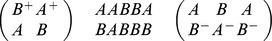. There are two CNV regions, 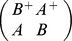 and 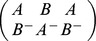. Based on equation (5), HaplotypeCN provides estimated copy numbers of A and B allele on each locus in the first CNV region as A(0.96, 2.31) and B(1.64, 1.15). The allele specific copy numbers for the second CNV region are A(0.92, 0.02, 1.06) and B(0.001, 0.87, 0.11). We will attach the copy numbers for either haplotype as 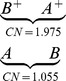 and 
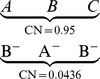
 by averaging the allelic specific copy numbers on the same haplotype. The average copy numbers for the first CNV region are 1.975 and 1.055. The copy numbers are 0.95 and 0.0436 for the second CNV region.

As mentioned above, there are two heuristic rules embedded in our algorithm. One is to constrain the states within the group of the shortest Mahalanobis distance, and the other is to adopt the shaded region in Figure 5. To evaluate the effect of either step, we conducted a comparison on chromosome 22 in the benchmark data of McCarroll’s. There are four conditions considered in the comparison. The concordance rates for the full model (36 genotypic states) with shaded region is 0.472 (26/55), and the concordance rate for the full model without shaded region is 0.44 (35/79). The concordance rates with and without the shaded region under the constraint of the shortest-distance groups are 0.47 (24/51) and 0.469 (31/66), respectively. Although the full model with shaded region led to the best result, it spent too much time and memory. Hence, the reduced model with shaded region strategy is recommended.

### Real Data Set

The 270 HapMap samples were genotyped with Affymetrix 6.0 arrays. The set consists of 30 Caucasian father-mother-child trios (CEU), 30 Yoruba trios (YRI), 45 unrelated Japanese individuals (JPT) and 45 unrelated Han Chinese (CHB). The allelic intensity for each SNP locus was summarized with the Birdseed algorithm. Although we choose Birdseed to demonstrate our algorithm, any algorithm that provides signals for both A and B alleles performs the same job.

We follow most relevant studies to compare the accuracy of the segmentation regions and the copy number estimates. If the segmentation and the copy number estimation are more favorable than or at least as favorable as the most popular algorithms that provide only standard information, the additional message of haplotype phasing can then be integrated in the association study with high confidence. Similar to other studies [36], we used two published reports as our benchmark data. They both record detailed copy number events on the HapMap individuals. Although the CNV events derived from those two reports were not comprehensive and were not experimentally validated, they were treated as high-confidence datasets to evaluate the six algorithms.

The first dataset is from the study of Kidd et al. [37]. The chromosome structural variations in eight samples from the HapMap project are detected by a fosmid end-sequence-pair (ESP) analysis. They provide chromosome copy number variation, insertion, and inversion events. The data were downloaded from the Database of Genomic Variant [38]. There are 1989 autosomal CNV events in at last one of the 8 HapMap individuals.

The second study we used is from McCarroll et al. [36]. They report copy number polymorphisms from the 270 HapMap individuals. Data were downloaded from the supplementary information in the original paper [36]. A total of 1292 copy number polymorphic (CNP) regions on autosomes were published for the 270 HapMap individuals. All the published events are more likely to be copy number polymorphisms (CNP) because no common diseases have been indicated for those individuals, and all the reported regions are observed in multiple unrelated samples. The data provide not only the regions, but also the sample-specific integer copy numbers on each CNP region of the 270 HapMap samples.

The algorithms we compared in this study include QuantiSNP, PennCNV, GenoCNV, COKGEN and cnvHap. Some of them were designed originally for the Illumina format. Hence, our first step involves using PennCNV-Affy (http://www.openbioinformatics.org/penncnv/penncnv_tutorial_affy_gw6.html) to extract LRR and BAF from Affymetrix SNP 6.0 array for the input files of QuantiSNP, PennCNV, GenoCNV and cnvHap. To generate canonical genotype clusters required for PennCNV-Affy, the 270 HapMap samples are used for training. QuantiSNP version 2.3 was used to processes the files individually on the HapMap 270 samples. GenoCN was downloaded as the R package version 1.06. The cnvHap was downloaded as the java package version v1.033. The input of PennCNV, COKGEN, GenoCNV and QuantiSNP includes both copy number (CN) probes and SNP probes. By contrast, HaplotypeCN and cnvHap make use of only SNP probes.

### Simulation Data

To better confirm our observation from the above analysis and to evaluate the haplotype phasing, we provide simulation study to support our conclusions. We consider only those methods that can predict CNVs without CN probes. PennCNV-SNP is thus included to make a fair comparison. The simulated phase-known datasets were generated from 44 CEU males, 45 CHB/JPT males, and 49 YRI males using only the X chromosome. We first excluded the pseudoautosomal regions, PAR1 and PAR2, which are homologous regions to the Y chromosome[39]. The remaining region forms a haplotype that can be easily detected and correctly constructed from most popular CNV calling software. By sampling with replacement, two chromosomes from different individuals were randomly chosen to make a new diploid within each ethnic group. The procedure created 90 pairs of chromosomes for each of the three ethnic groups. Since some chromosomes have acquired amplification or deletion, our pairing strategy mimics the copy number variation patterns of autosomal chromosomes as a consequence of random mating. There were 448 CNV events in the simulated data. For each pair of chromosomes selected, their A allele summaries derived from PennCNV-Affy were added up and so were the B allele summaries. The next step was to transform them into Log R Ratio (LRR) and B Allele Frequency (BAF) for each SNP site. The details of the simulation are provided in File S2.

**Figure 6:**
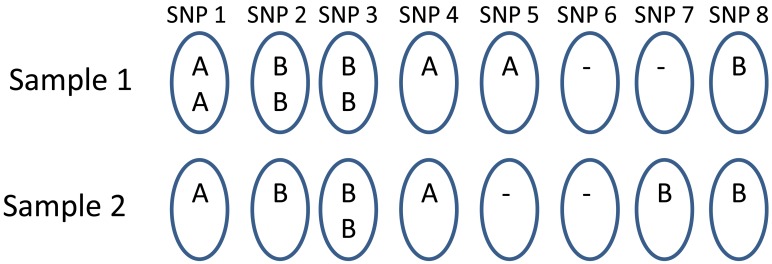
A toy example of simulation. The two male samples in the figure both provide their X chromosomes with their original amplifications or deletions. The chromosome from sample 1 carries amplifications from SNP1 to SNP3 and deletions at SNP6 and SNP7. The chromosome from sample 2 carries amplification at SNP3 and deletions at SNP5 and SNP6.

In Figure 6, we use a toy example of eight SNPs to explain it. The original data was from the male X chromosome so each sample consists of only one chromosome. Sample 1 is a chromosome amplified from SNP1 to SNP3 and sample 2 is a chromosome amplified at SNP3 only. When the two chromosomes are paired together to form a diploid sample, the amplified region is still SNP1 to SNP3. The total copy numbers for the three SNPs are 3, 3, and 4 in the sequential order. However, we want to be able to tear apart different amplified regions in either chromosome in our method. The proposed method will declare SNP1 to SNP3 jointly to be an amplified region while the copy numbers will be estimated separately. Similarly, the second CNV region consists of SNP5, 6 and 7. The total copy number in this case would be 1, 0, and 1 for the three SNP sites. We evaluate the accuracy in four levels:

1. The number of predicted CNV regions that overlap with the 448 simulated events. PennCNV-SNP, HaplotypeCN and cnvHap were evaluated.
2. The number of predicted CNV regions with correct total copy numbers. PennCNV-SNP, HaplotypeCN and cnvHap were evaluated.
3. The number of SNP sites with correct allele-specific copy numbers. SNP sites in the predicted CNV regions of correct total copy numbers were evaluated for HaplotypeCN and cnvHap.
4. The number of SNP sites with correct haplotype phasing. SNP sites in the predicted CNV regions of correct allelic specific copy numbers were evaluated for HaplotypeCN and cnvHap.

All of the above criterions were compared by calculating the ratio between those numbers and the total numbers of detection. They are represented as Rregion, Rtotal, Rspecific and Rhap, respectively, in the following discussion.

## Results

In this section, we first summarize the predicted CNV calls from the output of the six algorithms. We then compare the sensitivity according to the consistency with the published CNV regions, from Kidd et al. (2008) and McCarroll. We also used the deletion events in the 1000 genome project as the benchmark. The results are roughly parallel to what we observed with the above two sets and are reported in Table S1 in File S1 document. Simulation results will be provided in the end to compare the phasing accuracy.

### Summary Statistics of the CNV Calls

After analyzing the Affymetrix SNP array 6.0 data from HapMap 270 individuals, we summarize the outputs of these CNV calls in Table S2 in File S3. HaplotypeCN and cnvHap use only the SNP probes to infer the haplotype phases so we did an extra experiment on PennCNV with only SNP probes as input. It is termed PennCNV-SNP in this report. Since HaplotypeCN provides fractional allelic copy numbers, and any number between 0.5 and 1.5 will be rounded to 1 for comparison, a normal diploid could have an estimated total copy number from 1.5 to 2.5. HaplotypeCN will report CNV events when the estimated copy numbers are greater than 3 or smaller than 1. Table S2 in File S3 includes the median number of CNV calls, the median size of CNV calls, and the median number of markers within each CNV call per sample. We find that GenoCNV tends to obtain a lot more events with shorter length relative to the other four methods. HaplotypeCN is more conservative of calling CNVs, and the predicted regions are of comparable length to cnvHap.

The statistics are reported for different ethnic groups separately. Research has shown that the gene diversity of African population is larger than the other two ethnic groups. Table S2 in File S3 exhibits more CNV calls with shorter regions in YRI population relative to other ethnic groups, regardless of which algorithm we use.

### Comparing the CNV Calls Detected in the Eight Samples According to the Data of Kidd et al. (2008)

In 2008, Kidd et al. [37] published the structural variation events in eight samples detected by a clone-based method. We treat the published events as benchmark CNVs to match the events predicted by the six algorithms in this study. A predicted region is defined as a concordant event if it overlaps a benchmark CNV region. We first compare the proportion of true positives among the predicted events in Table 3 across the four algorithms that use both SNP probes and CN probes. The total number of predicted events varies considerably across the four algorithms. QuantiSNP and GenoCNV derive more than a thousand calls for the eight samples, while COKGEN and PennCNV are at the scale of a few hundreds. PennCNV has the best concordance rate among the four in this and other comparisons we do not show here. Hence, we compared PennCNV-SNP with cnvHap and the proposed HaplotypeCN in Table 4. It is worth some note here that data from Kidd et al. consist of mostly large CNVs and hence might benefit methods that tend to detect long stretches.

**Table 3:** **Comparison of concordant events across the four algorithms using the benchmark events published by Kidd et al (2008).**

Method	Concordance Rate^1^	Sensitivity
PennCNV	**212/584 (36.3%)**	212/1989 (10.65%)
COKGEN	151/440 (34.3%)	151/1989 (7.59%)
GenoCNV	550/6002 (9.2%)	550/1989 (27.65%)
QuantiSNP	373/1617 (23.1%)	373/1989 (18.75%)

1 Concordance Rate = number of concordant events/number of predicted events. The concordant events refer to the predicted segments that overlap with the benchmark events for least one SNP loci.

**Table 4:** **Comparison of concordant events across the three algorithms using the benchmark events published by Kidd et al (2008).**

Method	Concordance Rate^1^	Sensitivity
HaplotypeCN	**24/63 (38.1%)**	24/1746 (1.37%)
PennCNV-SNP	61/170 (35.9%)	61/1746 (3.49%)
cnvHap	89/365 (24.4%)	89/1746 (5.10%)

1 Concordance Rate = number of concordant events/number of predicted events. The concordant events refer to the predicted segments that overlap with the benchmark events for least one SNP loci.

### Comparing the CNV Calls with the Benchmark Events Published by McCarroll et al. (2008)

McCarroll et al. published 1292 autosomal CNP regions for 270 HapMap individuals. It is the second benchmark set in our comparison. The concordance rates for the four algorithms that use both SNP and CN probes are compared in Table 5 and the other three are listed in Table 6. The conclusion is similar to what were observed in the previous benchmark data. HaplotypeCN has the highest concordance rate. The results provide the evidence that the regions detected by HaplotypeCN are as accurate as most popular packages.

**Table 5:** **Comparison of concordant events across the four algorithms using the benchmark events published by McCarroll et al (2008).**

Method	Concordance Rate^1^	Sensitivity
PennCNV	12166/21069 (57.7%)	12166/42017 (28.83%)
COKGEN	8930/14624 (61.1%)	8930/42017 (31.65%)
GenoCNV	29866/212398 (14.1%)	29866/42017 (71.08%)
QuantiSNP	19665/53800 (36.6%)	19665/42017 (46.80%)

1 Concordance Rate = number of concordant events/number of predicted events. The concordant events refer to the predicted segments that overlap with the benchmark events for least one SNP loci.

**Table 6:** **Comparison of concordant events across the three algorithms using only SNP probes events published by McCarroll et al (2008).**

Method	Concordance Rate^1^	Sensitivity
HaplotypeCN	**1235/2586 (47.76%)**	1235/22844 (5.41%)
PennCNV-SNP	3206/6759 (47.43%)	3206/22844 (14.03%)
cnvHap	4087/22702 (18.0%)	4087/22844 (17.89%)

1 Concordance Rate = number of concordant events/number of predicted events. The concordant events refer to the predicted segments that overlap with the benchmark events for least one SNP loci.

The results from Table 3 to Table 6 convey the critical message that CNV detection based on array platforms still has considerable space for improvement. The proposed method performs comparably well to those popular packages for the CNV detection, while it provides extra information of phased haplotypes. There is no experimentally derived benchmark data to compare the phasing accuracy. Therefore, we conducted a simulation study as follows.

### Comparing the CNV Calls with Simulation Data

The detection accuracies for CNV regions and copy number estimation across the three algorithms are shown in Table 7. Both HaplotypeCN and cnvHap provide allele specific copy numbers for individual sites. The cnvHap and HaplotypeCN are both constructed under the HMM framework. cnvHap adopts a two-stage procedure to segment the region first and then generate the CNV haplotype. It provides discrete copy numbers while our HaplotypeCN provide fractional copy numbers that can better address somatic mutations. The estimated allelic copy numbers of HaplotypeCN were rounded to the closest integer for comparison. The total copy number was averaged across SNP sites of the same region. The number was also rounded to the closest integer to derive Rtotal. To be consistent to the above comparison, the number between 1 and 3 was deemed a normal event. In Table 7, HaplotypeCN predicted 113 CNV events while 110 were true. Among the 110 correct regions, 102 were resulted in the right total copy numbers. There were 938 SNPs sites in those 102 regions and 800 of them got the right allelic copy numbers for both A and B. Although HaplotypeCN has the least number of CNV calls, its detection accuracy is the best.

**Table 7:** **Comparison across the three algorithms in terms of overlapping regions, total copy numbers and allele specific copy numbers.**

Method	Rregion	Rtotal	Rspecific
HaplotypeCN	**97.345% (110/113)**	**92.72% (102/110)**	**85.29% (800/938)**
PennCNV-SNP	91.163% (196/215)	92.85% (182/196)	NA
cnvHap	64.305% (236/367)	85.16% (201/236)	72.25% (2382/3297)

Haplotype phasing is a novel feature that is only provided in cnvHap and HaplotypeCN. For SNPs with the right allelic copy numbers, the phasing accuracy is compared. Among the 800 SNP sites found to have correct allelic copy numbers in HaplotypeCN, 645 were arranged in the right haplotype phase. The accuracy of haplotype phasing (Rhap) is 80.63% (645/800), better than the 77.04% (1835/2382) of cnvHap.

### Application to Oral Cancer Samples

We demonstrate the detection of allelic copy numbers on patients with oral cavity squamous cell carcinoma (OSCC). There were a total of 112 OSCC samples genotyped with the Affymetrix SNP Array 6.0 platform, and the copy number amplification events on chromosome 8q22.2 to 8q24.3 were reported to be associated with extracapsular spread and second primary tumor development [40]. The data are available in Gene Expression Omnibus database under the accession number GSE25104. We evaluated the allelic copy numbers again with HaplotypeCN on the same region and associated the new measures with various clinical outcomes. Among the copy number alteration regions detected in our analysis, we select the region with the most events to demonstrate the results. The fractional copy numbers were associated with the time to relapse using Cox regression. The hazard ratio is 2.992 with p-value 0.017 compared to the hazard ratio 2.806 with p-value 0.039 in the original study. In addition, the copy number difference between patients with and without extra capsular spread is significant with p-value 0.016 using t-test compared to the p-value 0.028 using chi-squared test in the previous study. These results were consistent with the previous study on which traditional Hidden Markov Model (HMM) were performed [40] and both showed better significance. The prognostic stratification of patients based on our copy number measurements may facilitate risk-adapted management of OSCC patients.

## Conclusions

We presented a copy number alteration detection method that was successfully applied to the whole genome genotyping arrays. The HMM-based algorithm can provide fractional copy numbers and reconstruct the allelic haplotypes within the regions of copy number alteration. The quantitative outputs provide refined information of the status of the sample. This is especially critical for the assessment of cancer samples that are extremely heterogeneous with different proportions of cells acquiring different somatic mutations. Hence, understanding the effect of specific allele with numeric association on various clinical measurements provides higher statistical power for the biomarker detection. The clinical association carried out with our fractional estimate of copy numbers in the cancer samples provides better detection power than that with integer copy number states.

The performance of HaplotypeCN in terms of detection of copy number alteration regions was demonstrated on the 270 HapMap samples with benchmark events from two published studies. The accuracy of haplotype phasing was also demonstrated on the simulation data. Our concordance rates are comparably well to the most popular tools in the genomewide summary even though we used considerably less information than all the other methods. In addition, HaplotypeCN provides the copy number haplotypes capable of inferring the chromosome that is more susceptible to aberrant recombination events. Chromosome cut points are crucial and may be guided by certain alleles around the segmentation point. Hence, it is of major interest for biologists to clarify which chromosome acquires the amplification or deletion events.

The proposed algorithm requires the bi-allelic information to type the copy number genotypes as well as haplotypes. Because most genotyping arrays include a large number of random probes designed for copy number detection only, they do not carry allelic information and are not integrated into our current study model. A potential remedy involves integrating a hypothetical second allele composed of the same probe intensity and assigned a fixed genotype in the recognition process. We are currently working on this particular approach. This study does not aim at a comprehensive comparison for all CNV detection schemes and HaplotypeCN is not the best one in terms of the detection accuracy among our comparison. Our purpose was to provide a tool with relatively good performance on the detection of CNV regions while at the same time provide haplotype phasing, which is not a common function in other CNV detection tools.

### Availability

HaplotypeCN is available from the following website:

http://www.stat.nthu.edu.tw/~wphsieh/HaplotypeCN.htm

## Supporting Information

File S1 **Comparing the CNV calls with the benchmark events published by 1000 genome project.**
(DOCX)

File S2 **Simulation of the LRR and BAF data.**
(DOCX)

File S3 **Summary statistics for the CNV calls.**
(DOCX)

File S4 **Performance of the PSCN algorithm.**
(DOCX)
