# Supplementary material for: HaplotypeCN: Copy Number Haplotype Inference with Hidden Markov Model and Localized Haplotype Clustering
Source: PLoS One. 2014 May 21;9(5):e96841. doi: 10.1371/journal.pone.0096841 (PMC4029584; doi:10.1371/journal.pone.0096841)
Supplement: File S1 — Comparing the CNV calls with the benchmark events published by 1000 genome project. (DOCX) [file pone.0096841.s001.docx]

**Supporting Information S1:**

**Comparing the CNV calls with the benchmark events published by 1000 genome project**

The 1000 genomes project provides deletion events from samples sequenced at low coverage. Not all of the 270 Hapmap individuals were covered by the 1000 genomes project. According to the latest release of the 1000 genomes project (<ftp://ftp-trace.ncbi.nih.gov/1000genomes/ftp/>), 34 CEU, 80 CHB and 38 YRI individuals are included and the total number of deletion events reported for CEU, CHB and YRI are 17313, 37975 and 25748, respectively. This is also a potential benchmark set for our evaluation. The results for comparison are shown in Supplementary Table 1. The table reports the proportion of deletion regions among all the copy number variation events detected, and the concordance rate as the proportion of true positives among the predicted events. As a result, most of the benchmark events were not detected by any of the three methods, and all three methods detected a lot more regions that were not included in the benchmark. It might be caused by the low coverage of the sequencing project or caused by differential detection preference between the two technologies. The relative performance still holds compared to results with data from Kidd and McCarroll.

**Table S1**- Comparison of concordant events across the three algorithms using the benchmark events from 1000 genome

| Method | CEU | | CHB | | YRI | |
| --- | --- | --- | --- | --- | --- | --- |
|  | Proportion of deletion | Concordance | Proportion of deletion | Concordance | Proportion of deletion | Concordance |
| HaplotypeCN | 163/265 (61.51%) | 8/163 (4.91%) | 454/959 (47.35%) | 23/454 (5.1%) | 264/351 (75.21%) | 6/264 (2.3%) |
| PennCNV-SNP | 754/1107 (68.11%) | 61/754 (8.09%) | 1770/2416 (73.26%) | 86/1770 (4.86%) | 1086/1453 (74.74%) | 46/1086 (4.24%) |
| cnvHap | 694/1435 (48.36%) | 31/694 (4.47%) | 3158/5328 (59.27%) | 53/3158 (1.68%) | 1594/2536 (62.85%) | 20/1594 (1.25%) |
