# Supplementary material for: HaplotypeCN: Copy Number Haplotype Inference with Hidden Markov Model and Localized Haplotype Clustering
Source: PLoS One. 2014 May 21;9(5):e96841. doi: 10.1371/journal.pone.0096841 (PMC4029584; doi:10.1371/journal.pone.0096841)
Supplement: File S2 — Simulation of the LRR and BAF data. (DOCX) [file pone.0096841.s002.docx]

**Supporting Information S2:**

**Simulation of the LRR and BAF data**

Here are the step-by-step details of our simulation procedure:

Step 1: Use the PennCNV-Affy package to transform the CEL files to the intensity summary of A and B alleles.

Step 2: Choose two people at random from the 43 CEU males to generate a diploid from their X chromosomes. This step was realized by adding up their A and B intensity separately to create a new summary file. There are in total 90 samples generated in the simulation set.

Step 3: Derive the model files, confidence files and genotype files from the summary files in Step 2 by the Birdseed algorithm. Birdseed will also provide a summary file for the A and B allele intensity.

Step 4: Use the PennCNV-Affy package again to transform the A and B allele intensities to LRR and BAF.
