# Supplementary material for: HaplotypeCN: Copy Number Haplotype Inference with Hidden Markov Model and Localized Haplotype Clustering
Source: PLoS One. 2014 May 21;9(5):e96841. doi: 10.1371/journal.pone.0096841 (PMC4029584; doi:10.1371/journal.pone.0096841)
Supplement: File S3 — Summary statistics for the CNV calls. (DOCX) [file pone.0096841.s003.docx]

**Supporting Information S3:**

**Summary statistics for the CNV calls**

Supplementary Table 2 includes the median number of CNV calls, the median size of CNV calls, and the median number of markers within each CNV call per sample.

**Table S2** - Summary statistics for the CNV calls

| Algorithm | population | Median number of CNV calls for each sample |  | Median size of CNV calls in kb |  | Median number of markers in each CNV call^1^ |  |
| --- | --- | --- | --- | --- | --- | --- | --- |
| **HaplotypeCN** | Total  CEU  YRI  CHB+JPT | 8  8  8  7 |  | 8.85  8.95  5.80  10.04 |  | 5  5  5  4 |  |
| **cnvHap^2^** | Total  CEU  YRI  CHB+JPT | 37.5  32  47  33.5 |  | 7.38  4.26  5.76  11.55 |  | 4  4  4  5 |  |
| **PennCNV-SNP** | Total  CEU  YRI  CHB+JPT | 20  19  23  19 |  | 20.45  20.97  13.72  36.9 |  | 7  7  6  7.5 |  |
|  | Total | 77 |  | 19.01 |  | 25 |  |
| **PennCNV** | CEU | 76.5 |  | 18.33 |  | 25 |  |
|  | YRI | 80.5 |  | 16.23 |  | 25 |  |
|  | CHB+JPT | 74 |  | 22.01 |  | 25 |  |
|  | Total | 54 |  | 24.09 |  | 27 |  |
| **COKGEN** | CEU | 54 |  | 25.07 |  | 28 |  |
|  | YRI | 59 |  | 21.85 |  | 27 |  |
|  | CHB+JPT | 50 |  | 24.87 |  | 26 |  |
|  | Total | 527 |  | 3.395 |  | 5 |  |
| **GenoCNV** | CEU | 431.5 |  | 3.552 |  | 5 |  |
|  | YRI | 866 |  | 2.936 |  | 4 |  |
|  | CHB+JPT | 494 |  | 3.903 |  | 5 |  |
|  | Total | 201.5 |  | 5.152 |  | 7 |  |
| **QuantiSNP** | CEU | 197 |  | 5.473 |  | 8 |  |
|  | YRI | 222 |  | 5.03 |  | 7 |  |
|  | CHB+JPT | 194 |  | 5.054 |  | 7 |  |

^1^Markers include both SNP and CN probes.

^2^The reported regions of cnvHap were removed if consisting of only one SNP.
