# Supplementary material for: HaplotypeCN: Copy Number Haplotype Inference with Hidden Markov Model and Localized Haplotype Clustering
Source: PLoS One. 2014 May 21;9(5):e96841. doi: 10.1371/journal.pone.0096841 (PMC4029584; doi:10.1371/journal.pone.0096841)
Supplement: File S4 — Performance of the PSCN algorithm. (DOCX) [file pone.0096841.s004.docx]

**Supporting Information S4:**

**Performance of the PSCN algorithm**

PSCN can infer the parent specific copy numbers. That is, they provide the fractional copy number for each one of the two chromosomes and two allele states (ex. Gain/Loss, Gain/Normal, Gain/Gain, Loss/Normal, Loss/Loss) for the CNV regions detected. We ran chromosome 1 to 22 for CEU, CHB and YRI. PSCN detected 237,951 CNV regions, and the proportion of Gain/Loss is as high as 86.4% (205,652/237,951). Those are potential LOH regions, and our benchmark data do not include any LOH events. If the Gain/Loss events are removed from their output, there are 30,255 events left. We matched the results of PSCN with the benchmark events published by McCarroll et al (2008). When PSCN derives a Gain/Normal or Gain/Gain event that matches to an amplification event of the benchmark data, it is counted as a concordant event. When PSCN derives a Loss/Normal or Loss/Loss event that matches to a deletion event of the benchmark data, it is also counted as a concordant event. Hence, the concordance rate is 10.12% (3063/30,255). If we consider all the 237,951 events, the Gain/Loss events could also match to the amplification or deletion events of the benchmark data. Hence, we set thresholds on the total estimated copy numbers provided by PSCN to classify each event as deletion, LOH or amplification. The results are listed in the following Supplementary Table 3. The concordance rates are really low no matter what rules we applied and which ethnic group is considered. Based on the above observations, PSCN cannot handle a general purpose CNV detection with a reasonable accuracy. The extension from the LOH detection to the general purpose CNV detection is not so straightforward.

Supplementary Figure 1 to Figure 3 are the histograms of the estimated fractional copy numbers added from both haplotypes based on the outputs of PSCN for different ethinic groups. They are centered around 2 and suggest detections of LOH events. Supplementary Figure 4 to Figure 6 are the histograms of the estimated fractional copy numbers for the PSCN events that overlap with the benchmark events published by McCarroll et al. There is a heavy left tail in each of the figure. That is, they have better ability to detect the deletion events than the amplification events. Overall speaking, the estimated copy numbers do not deviate from 2 a lot while the benchmark events are mostly with copy number 1 or 3. It seems to us that their algorithm tends to limit the detection within copy neutral events.

We also applied PSCN on the simulation data. PSCN detected 801 CNV regions and the proportion of the Gain/Loss is 93.5% (749/801). Note that our simulation did not produce any copy neutral events and there seems to be over-detection of LOH events. If we do not consider those Gain/Loss events, there are 16 events overlapped with our simulated events. The rate for overlapping regions is 30.7%(16/52). We need to know the allele-specific copy number and haplotype phasing for the reported CNV regions. Their package does not provide the function to output the accompanied alleles with the reported copy numbers so we traced their R codes to output this information. This is the information required to form the haplotypes on CNV regions. Among the 16 regions of their detection, there are 11 matched to the correct allele specific copy numbers we simulated. The accuracy for allele specific copy number is 68.75% (11/16). Those 11 events consist of 2,325 SNP loci. If we count the correct arrangement of alleles on either chromosome for each locus, there are 468 loci matched to our simulation, and the accuracy for the right haplotype phase is 20.1% (468/2325).

**Table S3** - Comparison of concordant events across the PSCN algorithm using the benchmark events published by McCarroll et al (2008)

| Set | Concordance Rate^1^ when the CNVs are called for the estimated copy number ≥2.5 or ≤1.5 | Concordance Rate^1^ when the CNVs are called for the estimated copy number ≥2.4 or ≤1.6 | Concordance Rate^1^ when the CNVs are called for the estimated copy number ≥2.3 or ≤1.7 | Concordance Rate^1^ when the CNVs are called for the estimated copy number ≥2.2 or ≤1.8 | Concordance Rate^1^ when the CNVs are called for the estimated copy number ≥2 or ≤2 |
| --- | --- | --- | --- | --- | --- |
| CEU | 0.6% (517/84232) | 0.8% (645/84232) | 0.9% (785/84232) | 1.1% (965/84232) | 2.6% (2198/84232) |
| CHB | 0.5% (489/100464) | 0.6% (650/100464) | 0.8% (806/100464) | 1.2% (989/100464) | 2.4% (2381/100464) |
| YRI | 1.0% (517/53255) | 1.2% (634/53255) | 1.4% (751/53255) | 1.7% (903/53255) | 3.7% (1968/53255) |

^1^Concordance Rate=number of concordant events/number of predicted events

**Figure S1-** **The distribution of the estimated copy numbers derived from PSCN for the CEU group.** The total copy numbers are added from the estimated copy numbers of both haplotypes and the histogram consists of 84,232 events.

**Figure S2-** **The distribution of the estimated copy numbers derived from PSCN for the CHB group.** The total copy numbers are added from the estimated copy numbers of both haplotypes and the histogram consists of 100,464 events.

**Figure S3- The distribution of the estimated copy numbers derived from PSCN for the YRI group.** The total copy numbers are added from the estimated copy numbers of both haplotypes and the histogram consists of 53,255 events.

**Figure S4- The distribution of the estimated copy numbers from the benchmark-matched events for the CEU group.** The events detected by PSCN are limited to those overlapping with the benchmark events. The total copy numbers are added from the estimated copy numbers of both haplotypes and the histogram consists of 2198 events.

**Figure S5- The distribution of the estimated copy numbers from the benchmark-matched events for the CHB group.** The events detected by PSCN are limited to those overlapping with the benchmark events. The total copy numbers are added from the estimated copy numbers of both haplotypes and the histogram consists of 2381 events.

**Figure S6- The distribution of the estimated copy numbers from the benchmark-matched events for the YRI group.** The events detected by PSCN are limited to those overlapping with the benchmark events. The total copy numbers are added from the estimated copy numbers of both haplotypes and the histogram consists of 1968 events.
